# Hyperbaric Oxygenation: Can It Be a Novel Supportive Method in Acute Kidney Injury? Data Obtained from Experimental Studies

**DOI:** 10.3390/cells13131119

**Published:** 2024-06-28

**Authors:** Sanjin Kovacevic, Nikola Mitovic, Predrag Brkic, Milan Ivanov, Maja Zivotic, Zoran Miloradovic, Jelena Nesovic Ostojic

**Affiliations:** 1 Department of Pathological Physiology, Faculty of Medicine, University of Belgrade, 11000 Belgrade, Serbia; sanjin.kovacevic@med.bg.ac.rs (S.K.); nikola.mitovic@med.bg.ac.rs (N.M.); 2 Department of Medical Physiology, Faculty of Medicine, University of Belgrade, 11000 Belgrade, Serbia; predrag.brkic@med.bg.ac.rs; 3 Institute for Medical Research, Department of Cardiovascular Physiology, National Institute of Republic of Serbia, University of Belgrade, 11000 Belgrade, Serbia; ivmilan@imi.bg.ac.rs (M.I.); zokim@imi.bg.ac.rs (Z.M.); 4 Institute of Pathology, Faculty of Medicine, University of Belgrade, 11000 Belgrade, Serbia; majajoker@gmail.com

**Keywords:** hyperbaric oxygen, acute kidney injury, cellular physiology

## Abstract

Despite constant achievements in treatment, acute kidney injury (AKI) remains a significant public health problem and a cause of mortality in the human population. In developed countries, AKI is a significant and frequent hospital complication, especially among patients admitted to intensive care units, where mortality rates can reach up to 50%. In addition, AKI has been implicated as an independent risk factor for the development of chronic kidney disease. Hyperbaric oxygenation (HBO) has been used as a primary or adjunctive therapy for the past 50 years, both in experimental and clinical studies. HBO is a treatment in which the patient is occasionally exposed to 100% oxygen at a pressure greater than atmospheric pressure at sea level. However, despite decades of extensive research, the potentially beneficial effects of this therapeutic approach are still not fully understood, although many potential mechanisms have been proposed, such as antioxidative, anti-inflammatory, anti-apoptotic, etc. Furthermore, the low cost and insignificant adverse events make HBO a potentially important strategy in the prevention and treatment of different diseases. Considering all of this, this review highlights the potential role of HBO in maintaining cellular homeostasis disrupted due to AKI, caused in different experimental models.

## 1. Introduction

Acute kidney injury (AKI), previously known as acute renal failure, is a clinical syndrome characterized by a rapid decrease in renal excretory function, with a decreased glomerular filtration rate (GFR), followed by the accumulation of nitrogen metabolism products such as creatinine and urea and other metabolic waste products. Other common clinical features of AKI include oliguria, alteration of water and electrolyte balance, alterations of acid–base balance, etc. [1].

In terms of global mortality, AKI significantly surpasses mortality rates associated with heart failure, diabetes, and breast cancer. Despite advancements over the past 50 years, AKI-related mortality rates have remained persistently high. The incidence of AKI is followed by large differences between developing and developed countries [2]. In high-income countries, AKI is mostly a hospital-acquired condition, with the most common causes including renal ischemia, sepsis and nephrotoxic drugs. On the other hand, in low-income countries, AKI is mostly a consequence of community-acquired disease (infectious diseases, diarrhea, dehydration, animal venoms, etc.) [3]. In developing countries, considering numerous community-related causes, patients tend to be younger, with limited access to hospital treatment, while in developed countries, patients with AKI tend to be older, with multiple comorbidities. In hospitalized patients, AKI is most prevalent in intensive care units with its prevalence estimated to be up to 60% in critically ill patients. In this setting, AKI-related costs are very high, and prevention is difficult [2,3].

Risk factors for AKI include environmental (inadequate drinking water, control of infectious diseases and unavailable health care systems), socioeconomic factors related to the treatment and patients themselves. Patient-related factors can be modified (hypovolemia, hypertension, hypotension, anemia, hypoxia, and nephrotoxic drug usage) or nonmodified (chronic kidney disease (CKD)), heart failure, liver failure, diabetes, infections and sepsis). The most important risk factors for AKI are acute infections, sepsis, trauma, hypovolemia, old age, CKD, acute organ failures, major surgeries, hospitalization in the intensive care unit with exposure to nephrotoxic drugs and opportunistic infections, chemotherapy, urinary tract obstruction, etc. In the context of hospital-related risk factors, severe AKI occurs more frequently during major surgery procedures, bleeding, septic shock or drug toxicity in older patients with comorbidities [2].

Considering the AKI etiology, numerous conditions can cause AKI. Those factors are divided into three main categories: pre-renal, renal (intrinsic) and post-renal. In pre-renal AKI, hypoperfusion leads to decreased GFR, without direct harmful effects on the renal parenchyma, as a compensatory response to various extra-renal insults (hypovolemia, impaired cardiac function, systemic vasodilatation, and increased vascular resistance). However, if the hypoperfusion lasts, or if there is a more severe hypoperfusion, ischemic injury will occur with the development of acute tubular necrosis (ATN) and renal (intrinsic) AKI [3]. Renal (intrinsic) AKI occurs as a result of factors that are directly injurious to the renal parenchyma. ATN is the term used to designate AKI resulting from tubular damage, which is the most common type of intrinsic kidney injury. Intrinsic AKI can also result from glomerular injury (severe acute glomerulonephritis), vascular injury to intra-renal vessels and acute interstitial nephritis [3,4]. Ischemia and nephrotoxic substances cause direct ATN. Many pharmacological substances have a toxic effect on the tubules, and nephrotoxic ATN can be a complication of their use [5]. Intrinsic AKI is very often a complication of sepsis and severe rhabdomyolysis. Post-renal AKI rarely occurs due to acute obstruction of the urinary flow, which increases intra-tubular pressure and thus decreases GFR [3].

The pathophysiology of AKI is multifactorial and complex. Ischemia is a common cause of AKI, leading to a disruption in the supply of oxygen and essential nutrients to kidney cells, resulting in impaired removal of waste products. This imbalance between oxygen availability and demand within the tissue contributes to the accumulation of metabolic waste products. As a result of this imbalance, the tubular epithelial cells are injured, and if the damage is complex and severe, cell death occurs, either by the process of apoptosis or necrosis. Although increasing the oxygen supply in order to ameliorate AKI is a well-known strategy, recent studies have also focused on reducing oxygen demand as another way to maintain the supply–demand balance of oxygen when the supply is low. Experimental models have demonstrated that the inhibition of sodium reabsorption can alleviate AKI. Since the proximal tubules reabsorb approximately 65% of filtered sodium, consuming the largest portion of oxygen, potential therapeutics that have been examined include acetazolamide, dopamine, inhibitors of the renin–angiotensin II system, etc. [6]. The pathophysiological mechanisms of ischemic AKI are diverse and intricate and include hypoxic cell damage, increased levels of oxidative stress, hemodynamic alterations, inflammation, and endothelial and epithelial dysfunction [7]. In AKI due to sepsis, in addition to ischemia, deleterious effects on tubular endothelial cells occur by lysosomal enzymes and free radicals released from activated leukocytes, microvascular dysfunction, and proinflammatory cytokines secreted by tubular epithelial cells. In AKI caused by rhabdomyolysis, the released substances such as myoglobin and sarcoplasmic proteins, due to the necrosis of skeletal muscle cells, can be filtered through the glomeruli, leading to injury via different mechanisms, such as intratubular obstruction, renal vasoconstriction, inflammation and tubular damage associated with reactive oxygen species (ROS) production. Different medications, particularly nephrotoxic drugs, can also have toxic effects on the kidney as glomerular, interstitial and tubular cells come into contact with significant concentrations of medications and their metabolites, which can induce changes in kidney structure and function. Tubular epithelial cells are especially vulnerable to the toxic effects, considering their role in concentrating and reabsorbing glomerular filtrate, which exposes them to high levels of circulating drugs. Renal toxicity can be a result of hemodynamic changes, direct cell injury, inflammatory tissue injury and obstruction of renal excretion [3].

Hyperbaric oxygenation (HBO) has been used as a primary or adjunctive therapy for the past 50 years, both in experimental and clinical studies. HBO is a treatment in which the patient is occasionally exposed to 100% oxygen at a pressure greater than atmospheric pressure at sea level [8,9]. However, despite decades of extensive research, the potentially beneficial effects of this therapeutic approach are still not fully understood, although many potential mechanisms have been proposed [10]. In contrast to classical pharmacological forms of prevention and treatment, HBO seems to show certain advantages in ischemic injury, primarily because it offers a specific oxygen reservoir that can last up to several hours and is of great importance in the case of sudden ischemia and hypoxia, improving endothelial function and reducing local edema and inflammation. Also, of great importance is the fact that oxygen reaches the cells not only through the blood but also by diffusion from the interstitium, where it reaches a high concentration during hyperbaric oxygenation treatment, providing greater availability compared to any pharmacological agent. Also, HBO can directly affect gene expression, signal transduction, mitochondrial function, and cell apoptosis and induce the expression of antioxidant enzymes [11,12,13,14]. Furthermore, the low cost and insignificant adverse events make HBO a potentially important strategy in the prevention and treatment of different diseases [8,15].

Considering the fact that, to date, the management of AKI involves the elimination or treatment of the primary cause of established specific AKI and the maintenance of tissue homeostasis during its recovery, as well as that AKI prevention primarily targets risk factors or potential triggers [1], for AKI development, this systematic literature review aims to investigate the effects of hyperbaric oxygen, used as preconditioning or therapy, in various animal experimental models of AKI. Specifically, it focuses on assessing its impact on kidney tissue function and morphology, with a particular emphasis on cellular physiology.

### Molecular Mechanisms of Hyperbaric Oxygen

The beneficial effects of HBO are explained by an increase in the partial pressure of oxygen in tissue in hyperbaric conditions, even without hemoglobin contribution, but also by the activation of cellular signaling pathways in conditions of “controlled” oxidative stress, i.e., in conditions of “moderate” formation of ROS. Thus, HBO initiates different mechanisms in the organism, and it can be used to correct tissue hypoxia and chronic hypoxemia, but beneficial effects of HBO are also recognized in different pathological conditions including reperfusion injuries, necrosis and wound healing [9,16]. Currently, there are 14 approved indications for HBO therapy: air or gas embolism, acute thermal burn injury, carbon monoxide poisoning, carbon monoxide toxicity complicated by cyanide poisoning, central retinal artery occlusion, clostridial myositis and myonecrosis (gas gangrene), compromised grafts and flaps, crush injury, compartment syndrome and other acute traumatic ischemia, decompression sickness, delayed radiation injury (soft tissue and bony necrosis), enhancement of healing in selected problem wounds, idiopathic sudden sensorineural hearing loss, intracranial abscess, necrotizing soft tissue infections, refractory osteomyelitis and severe anemia [9].

Persistent tissue hypoxia can lead to organ dysfunction and functional disorders. In order to prevent such harmful effects, many cellular genes are expressed with the aim of promoting cellular protection and repair. Hypoxia-inducible factor 1 (HIF-1) is the main transcription factor that induces approximately 200 genes important for adapting cells to decreased oxygen values [12].

However, the entire spectrum of action of the HBO therapeutic approach is still not fully understood. In addition to the use of hyperbaric oxygenation as a therapeutic modality, experimental studies and clinical observations support the evidence that preventive treatment with hyperbaric oxygenation, applied in the presence of risk factors for the development of ischemia-reperfusion damage, has a protective effect. This protective impact is based on the effects of intermittent hyperoxia and can be interpreted through the phenomenon known as the “hypoxia–hyperoxia paradox” [17]. In short, a hyperoxic tissue environment is accompanied by more dissolved oxygen, followed by increased ROS and reactive nitrogen species (RNS) production. ROS and RNS can provoke many injurious effects on different macromolecules, including proteins, lipids, DNA and others, followed by reduced cellular functions. In order to prevent or escape such deleterious effects, the compensatory response is initiated, including an increase in antioxidant proteins (scavengers) to counteract the increased ROS. When returning to normoxia, levels of oxygen and ROS are normalized, but the scavenger activity remains high for a prolonged period. In fact, the half-life of scavengers exceeds that of ROS. Consequently, when hyperoxic exposure is repeated, the ratio of ROS to scavengers is reduced. This leads to a decrease in ROS levels and a higher level of active HIF entering the nucleus, facilitating the transcription of genes typically induced during hypoxia. Intriguingly, exposure to intermittent hyperoxia triggers many of the same mediators and cellular mechanisms as hypoxia. This suggests that cells perceive intermittent hyperoxia as a hypoxic state, leading to the promotion of cellular processes typically induced by HIF, but occurring under normoxic conditions [12].

On the other hand, cellular and molecular effects induced by HBO exposure are mediated by ROS-dependent cellular signaling. Additionally, the effects of ROS can be offset by the activity of nuclear factor erythroid 2-related factor 2, a transcription factor responsible for regulating the antioxidant response [12,18] However, the magnitude and duration of the antioxidant response depend on the applied oxygen dose [12,19].

In addition to antioxidant properties, current evidence suggests that HBO exerts antiapoptotic effects directly via the LC3II and Bcl-2 [11,20,21] pathways, but also via downregulation of caspase-mediated apoptosis [22]. On the other hand, HBO displays variable effects on proteins in the cytosol, LC3II, beclin 1 and m-TOR, involved in autophagy processes. Besides this, there are data that HBO downregulates ATG5 protein residing in the autophagosome membrane [11].

HBO has been observed to exhibit numerous anti-inflammatory effects [23,24], possibly attributed to its ability to modulate oxidative stress or directly influence the innate immune system [25]. Through various transcriptional factors such as HIF-1 and NF-kB, HBO significantly diminishes inflammatory cytokines [26,27]. HBO led to a reduction in pro-inflammatory cytokines and chemokines such as cyclooxygenase-2 [28]; interferons 1, 6, 8, and 10 [29,30]; TNF-α [31] and TGF-β [32]. A decrease in these factors results in reduced leukocyte migration, edema, and pain [11]. When microcirculation is impaired, HBO improves microvascular dynamics, in part by enhancing the production of nitric oxide that counteracts vasoconstriction [33]. In nerve tissue, HBO demonstrates a neuroprotective effect by activating protein kinase B and the Toll-like receptor 2/NF-κB signaling pathway. This activation leads to the downregulation of pro-inflammatory cytokines [34].

In addition to the mentioned effects, studies indicate that HBO also has a pro-angiogenic effect mediated by an increase in vascular endothelial growth factor, promoting the formation of new blood vessels. In wound healing, it affects the activity of nitric oxide synthase and synthesized nitric oxide mobilizes stem cells in bone marrow, promoting these processes. It also affects the proliferation and stimulation of stem cells, collagen synthesis and osteogenesis, with important antimicrobial effects [12].

## 2. Materials and Methods

**Figure 1:**
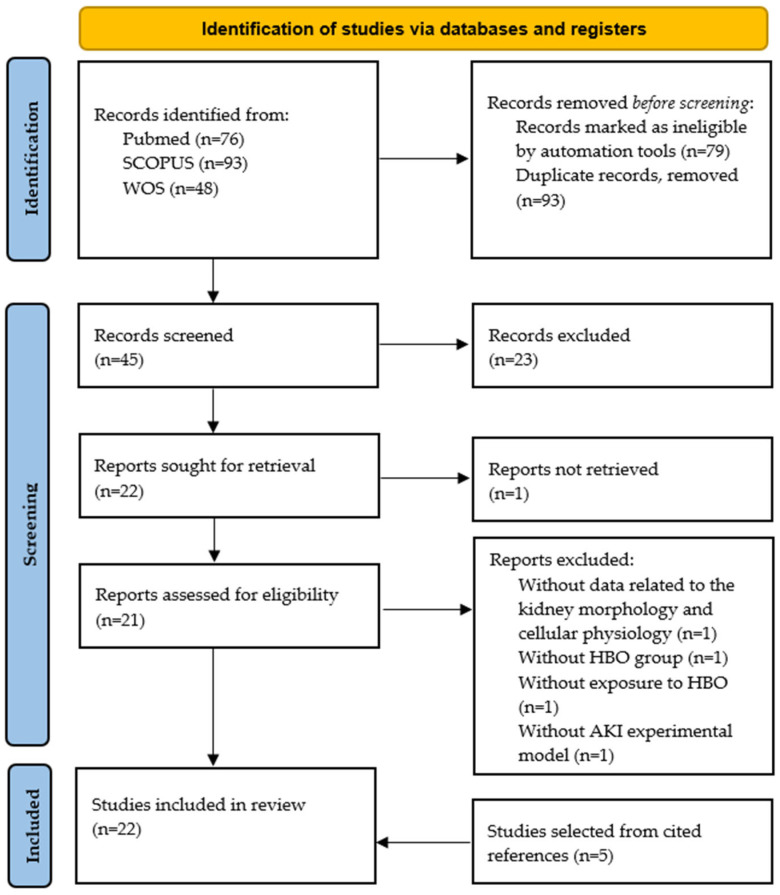
PRISMA flow diagram for the studies selection.

The literature review was conducted according to PRISMA guidelines [35]. In the publication search, three databases were used: Pubmed, Scopus and Web of Science. The literature search was conducted by two independent reviewers during July 2023, using the following keywords: acute kidney injury or acute renal failure and hyperbaric oxygen or hyperbaric oxygenation. The included research papers were original articles conducted on different experimental animal models of AKI in the last 20 years, written in English, that demonstrated the effects of hyperbaric oxygen on kidney tissue, cellular function and morphology, applied as preconditioning (before) or therapy (after) AKI induction, with a clearly described experimental and HBO exposure protocol (Figure 1). Information from selected articles was extracted and categorized based on their findings and then summarized to analyze and compare their respective results. This review was registered on the Open Science Framework Registries (OSF)—https://doi.org/10.17605/OSF.IO/ATNUX.

## 3. Effects of Hyperbaric Oxygen Preconditioning on Acute Kidney Injury

The main literature search findings are given in Table 1.

In all five studies, authors investigated the effects of HBO preconditioning on postischemic AKI [36,37,38,39,40]. In most of the mentioned studies, HBO preconditioning was applied 48 h before AKI induction and included four exposures that lasted 60 min during 2 days with two sessions per day (pressure exposure 2–2.5 ATA) [37,38,39,40]. On the other hand, in one study, HBO preconditioning was applied 30 min before surgery as a single session for 75 min with 2.8 ATA of pressure [36]. Two of the mentioned studies were conducted on normotensive Sprague–Dawley rats [36,37], two on spontaneously hypertensive rats [38,40], while one of the included studies used both spontaneously hypertensive and normotensive Wistar rats [39].

HBO preconditioning significantly improved renal morphology in all selected articles. Classic histopathological features of AKI, which include tubular cell necrosis, dilatation, the presence of PAS-positive casts and the loss of brush border, were diminished after HBO preconditioning [36,37,38,39,40].

In post-ischemic AKI, during ischemia, the renal microcirculation is disturbed, which results in reduced blood flow, hypoxia and oxidative stress. The metabolic and biochemical consequences of hypoxia are numerous, including anaerobic glycolysis, reversible and finally irreversible cell injury [41]. Renal ischemia-reperfusion injury is the main trigger for ROS production, and injured mitochondria are one of the main sources of ROS [42]. Xanthine oxidase as well as NADPH oxidase of activated leukocytes are additional sources of ROS formation. In the ischemic phase, ROS may influence the effects of vasodilators and vasoconstrictors and lead to an increase in renal vascular resistance. Superoxide anion promotes vasoconstriction and enhances the reactivity of angiotensin II and adenosine. Disturbed activity of antioxidant enzymes also contributes to the extent of renal injury, due to reduced removal of free radicals. Also, in animal models, it was demonstrated that postischemic AKI is followed by increased activity of HO-1, which is an important cytoprotective enzyme [43]. During the reperfusion phase, which is characterized by an inflammatory response mediated by leukocytes, additional oxidative stress and cellular damage occurs [41]. Re-establishing blood flow in ischemic tissue leads to an increase in ROS formation, which in turn contributes to further cell injury. ROS can act as secondary messengers and stimulate the activation of the transcription factor NF-κB, which causes the expression of adhesion molecules on endothelial cells. Leukocytes from the blood bind to the adhesion molecules on the endothelial cells, resulting in their activation and the release of newly formed ROS. Therefore, microvascular endothelium damage leads to the activation of endothelial cells with the expression of adhesion molecules on the cell surface, which promote the adhesion of leukocytes and platelets, leading to further changes in perfusion and oxygen delivery, and additional amplification of ROS generation, tissue injury and inflammatory response [7,43].

**Figure 2:**
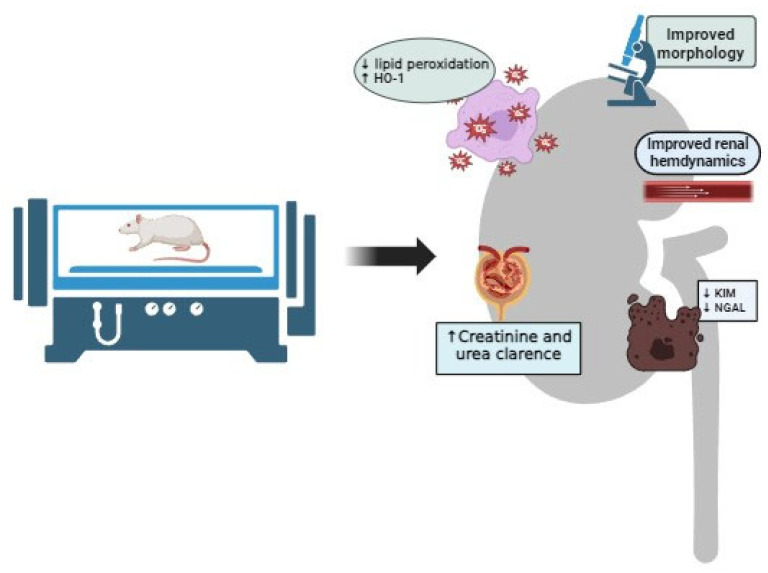
Summarized effects of HBO preconditioning in experimental post-ischemic AKI.

In selected articles, HBO preconditioning was capable of decreasing lipid peroxidation in kidney tissue [36,40] and upregulating cytoprotective HO-1 expression in kidney tissue [37,39,40]. However, Nesovic-Ostojic et al. [39] showed only significantly increased HO-1 expression in hypertensive rats. Taking into account the results of the effects of HBO preconditioning on kidney tissue, especially the effect related to the reduction in oxidative stress, it is not surprising that in the mentioned studies, an improvement in renal hemodynamic (increase in renal blood flow and reduction in renal vascular resistance) and function was noticed, primarily accompanied by a decrease in the plasma concentration of urea and creatinine or an increase in their clearances. In addition, these results were followed by a decrease in novel kidney biomarkers, such as plasma KIM-1, as well as a decrease in NGAL immunohistochemical expression in kidney tissue [37,38,39,40]. HBO preconditioning increased pro-apoptotic Bax, as well as anti-apoptotic Bcl-2 protein in kidney tissue [39]. Summarized effects of HBO preconditioning in experimental post-ischemic AKI are shown in Figure 2.

## 4. Effects of Hyperbaric Oxygen Therapy on Acute Kidney Injury

The main literature search findings are given in Table 2.

Considering the application of HBO as a therapeutic procedure after AKI induction, seventeen studies investigated its effects [44,45,46,47,48,49,50,51,52,53,54,55,56,57,58,59,60]. In six studies, an experimental model of post-ischemic AKI was used [44,45,46,47,48,49]. In addition to the previously mentioned studies, eleven other studies employed different experimental models to induce AKI. Two studies utilized a cisplatin-induced AKI model [50,51], two studies employed a glycerol-induced AKI model [52,53], two used a cyclosporine induced-nephrotoxicity model [54,55], one study was performed on a vancomycin-induced nephrotoxicity model [56], two used a gentamicin-induced nephrotoxicity model [57,58], one study utilized a sepsis-induced AKI model [59] and one used ischemia-reperfusion AKI after kidney transplantation [60].

### 4.1. Effects of Hyperbaric Oxygen Therapy on Post-Ischemic Acute Kidney Injury

In all selected studies [44,45,46,47,48,49], HBO therapy was applied the same day after AKI induction. In two studies, it was applied as a single session, 1 h [48] and 15 h after AKI induction [44], while in two studies, the HBO approach included two sessions per day for one day, 2 and 22 h [45] and 6 and 20 h [46] after AKI induction. One study examined the effects of two daily sessions [49], while one used exposure with five sessions for 5 days [47], with the first session 24 h after AKI induction. In all studies, pressure exposure was 2.5 ATA [44,45,46,47,48], except one, in which it was 2.4 ATA [49], with the session duration ranging from 60 min [44,46,47,48] to 90 min [45,49]. Five of the mentioned studies were conducted on Sprague–Dawley rats [44,45,46,48,49], while one of the included studies used Wistar rats [47].

Similar to HBO preconditioning, all studies reported a significant decrease in morphological alterations in kidney tissue caused by AKI after treatment with HBO [44,45,46,47,48,49]. By using the expression of specific markers, Ko et al. [49] showed that hyperbaric oxygenation has a beneficial effect on endothelial cell integrity, podocyte integrity and angiogenesis in post-ischemic AKI.

In post-ischemic AKI, apoptosis and necrosis can occur simultaneously with a certain dose dependence considering ischemia duration. Lower-degree ischemia, lasting for 15 min, leads to tubular epithelial cell apoptosis, without necrosis, while a longer duration of ischemia is associated with a higher degree of apoptosis and the appearance of necrosis. However, apoptosis and necrosis can be triggered by common, identical mechanisms, and other supporting factors determine whether cell lysis or programmed self-destruction will develop [43,61]. In addition to the beneficial effects on renal morphology and a lesser extent of morphological alterations, HBO in AKI also demonstrates anti-apoptotic properties [48,49], primarily through reduced expression of Bax, caspase 3, and cleaved PARP [49], accompanied by improvement in tubular regenerative capacity [48,49].

In post-ischemic AKI, damaged proximal tubular cells express MHC class II molecules on their surface and secrete various pro-inflammatory cytokines and chemokines, which are important in attracting and activating immune cells. The innate immune response is antigen non-specific and begins already 30 min after the onset of the reperfusion phase and includes direct alteration of tubular epithelial cells caused by activated neutrophil granulocytes, macrophages and NK cells, as well as the activation of the complement system. As the adaptive immune response is antigen-specific, it develops within a few hours of AKI onset and includes the activation, proliferation and interaction of T and B lymphocytes. Primary, neutrophil granulocytes are accumulated in the zone of the outer medulla, where the morphological alterations are the greatest. The initial interaction of these cells with the endothelium leads to the release of various products, such as myeloperoxidase, proteases, ROS and cytokines, increasing vascular permeability, tubular cell damage and further attraction of leukocytes. Additionally, the recruitment of monocytes and their differentiation into macrophages persists for seven days. Although the main function of macrophages is phagocytosis of damaged tissue material, they also play a key role in the adaptive immune response, primarily through antigen presentation to T lymphocytes [62].

**Figure 3:**
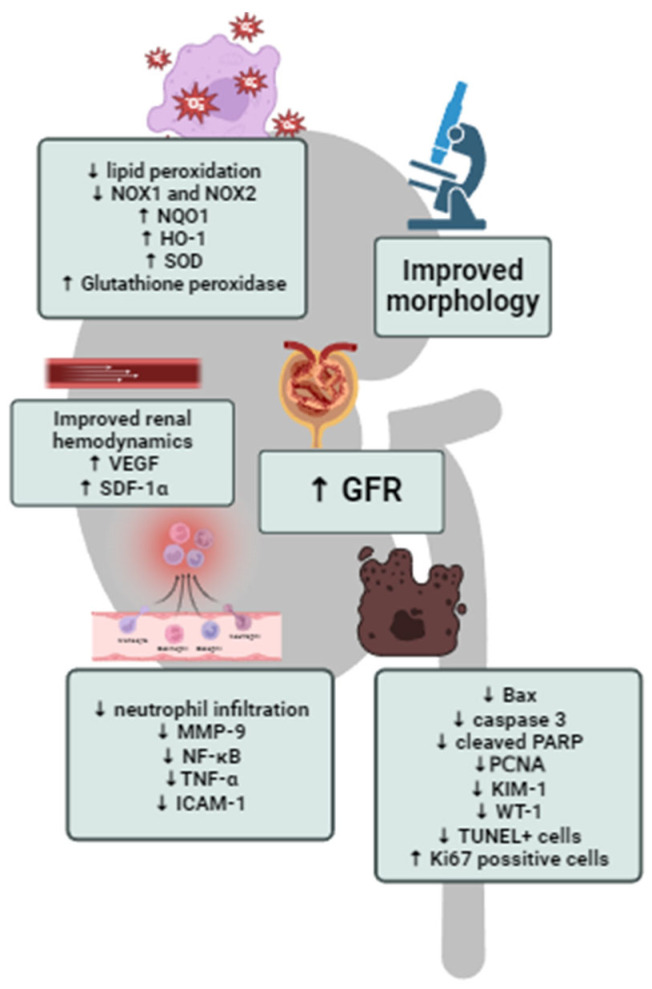
Summarized effects of HBO therapy in experimental post-ischemic AKI.

In the aforementioned studies, it was shown that HBO has an anti-inflammatory property, primarily by reducing neutrophil infiltration [44,47] and also decreasing expression of MMP-9, TNF-α, NF-κB and ICAM-1 [49]. Also, HBO, applied as a therapeutic approach, is capable of reducing oxidative stress levels, with decreased lipid peroxidation and NADPH oxidase 1 and 2 activity and improved activity of antioxidant enzymes in kidney tissue, such as SOD, glutathione reductase, HO-1 and NADPH quinone dehydrogenase [45,47,49]. Cytoprotective effects of hyperbaric oxygenation in experimental post-ischemic acute renal injury have resulted in significant improvement of renal function, primarily reflected through decreased plasma concentrations of creatinine and urea [44,46,49], increased GFR [45], and improved renal hemodynamic [45]. The summarized effects of HBO therapy in experimental post-ischemic AKI are shown in Figure 3.

### 4.2. Effects of Hyperbaric Oxygen Therapy on Cisplatin-Induced Acute Kidney Injury

Platinum-based drugs, like cisplatin, are extensively used in chemotherapy for treating solid tumors such as ovarian, head and neck, and testicular germ cell tumors. However, a recognized complication associated with cisplatin administration is AKI. As cisplatin accumulates over time, renal toxicity is dose-dependent, often causing dosage modification or discontinuation of therapy. Repeated AKI episodes can potentially lead to the development of CKD [63]. The pathophysiology and mechanisms involved in cisplatin-induced AKI are complex but not fully understood. However, a primary event associated with the nephrotoxic effect of cisplatin is platinum accumulation within tubular cells. Cisplatin accumulation triggers ROS production, followed by renal microvasculature vasoconstriction and renal ischemia. Also, it stimulates the inflammatory response, primarily due to increased production of TNF-α, with further inflammation, oxidative stress, vascular injury, and activation of apoptotic pathways. As a result of these processes, renal tubular injury occurs, either through apoptosis or necrosis, leading to renal tissue damage, followed by a decrease in GFR [64].

In two selected studies [50,51], HBO therapy was applied on the same day following AKI induction in Wistar albino rats. In one study, it was given as a single daily session right after cisplatin application for seven consecutive days [50]. In the other study, it was also given immediately after cisplatin administration, with one group receiving one session per day and another group receiving two sessions per day, over a period of six days [51]. Pressure exposure was consistent at 2.5 ATA, with each session lasting for 60 min in both studies [50,51].

In both presented studies, HBO treatment consisting of one daily session for either 6 or 7 days led to a significant improvement in the renal tissue morphology, reducing the level of alterations caused by cisplatin administration. This improvement resulted in decreased plasma concentrations of urea and creatinine [50,51], as well as an improvement in the clearance of these substances [50]. Interestingly, in this experimental model, the administration of HBO twice a day for 6 days (12 sessions in total) did not lead to morphological improvements or a decrease in plasma concentrations of urea and creatinine [51]. According to the findings from these studies, it is likely that a potentially favorable outcome observed in rats treated with HBO is antioxidative in nature. This is especially considering that HBO is capable of reducing lipid peroxidation in kidney tissue and improving the activity of antioxidative enzymes such as SOD and glutathione peroxidase. Despite the increased activity of these enzymes and reduced levels of lipid peroxidation following two daily HBO sessions, it is possible that two daily HBO sessions induced a ROS-independent pathway and aggravated the toxic effects of cisplatin [51].

It is possible that the pressure, duration, and frequency of HBO determine the cellular and subcellular effects of the treatment. A larger number of HBO therapy sessions has higher incidences of adverse effects [65]. One in vitro study showed an evident dose-dependent effect, where exposure to HBO at 2.5 ATA for 15 min was ineffective, while 30- and 60-min exposures increased the proliferation rate of cultured fibroblasts. On the other hand, 120 min of exposure resulted in increased apoptosis of cultured cells [66].

### 4.3. Effects of Hyperbaric Oxygen Therapy on Glycerol-Induced Acute Kidney Injury

Rhabdomyolysis is characterized by extensive necrosis of skeletal muscle, leading to the release of large quantities of intracellular contents from damaged muscle cells into the bloodstream. The development of rhabdomyolysis is associated with crush syndrome, exhaustive exercise, medications, infections and toxins, with AKI being one of the most severe complications [67]. The most commonly used experimental model for rhabdomyolysis-associated AKI in rats is a single intramuscular application of glycerol. Glycerol-induced AKI in rats is mediated by renal ischemia and myoglobin nephrotoxicity [68]. Rhabdomyolysis-associated AKI develops because of hypovolemia, tubular injury due to oxidative stress, renal vasoconstriction and tubular obstruction. Released myoglobin plays a fundamental role in the pathogenesis of rhabdomyolysis-associated AKI by increasing oxidative stress, inflammation, endothelial dysfunction, vasoconstriction, and cell injury. After myoglobin is released into circulation, it is filtered into the proximal tubule of the kidney. As myoglobin passes the nephron, it causes increased formation of ROS directly with other nephrotoxins released from the necrotic muscle, or by iron-mediated hydroxyl radical production. Also, myoglobin combines with Tamm–Horsfall protein and forms obstructive tubular casts. Myoglobin from the tubular system infiltrates into the interstitial space, causing an inflammatory reaction, primarily mediated by innate immune cells, such as neutrophils, macrophages and NK cells. This further triggers the release of mediators, including cytokines and chemokines, leading to amplification of inflammatory response and resulting in tubular injury [67,68,69].

In two specific experimental studies [52,53], HBO therapy was initiated on the same day after the induction of AKI. In the first study [52], HBO therapy was administered as a single daily session for two consecutive days, while in the second study [53], it was applied as two daily sessions, with one session occurring 2 h after AKI induction and the other session 18 h later. The pressure exposure differed between the studies, with one using 2.5 ATA for 90 min in Sprague–Dawley rats [52], and the other employing 2.4 ATA for 70 min in Wistar rats [53].

In one of the selected studies, HBO treatment showed a significant beneficial effect on kidney function with decreased urea and creatinine plasma levels, decreased urine sodium and fractional excretion of sodium and increased creatinine clearance. The authors confirmed the significant antioxidant effect of HBO, with decreased lipid peroxidation and increased SOD and catalase activity in kidney tissue. Significant morphological improvements in kidney tissue were followed by decreased tubular cell apoptosis and decreased PCNA expression [52]. Other study failed to demonstrate a beneficial effect of HBO in this experimental setting, with no differences in urea and creatinine values. Also, the authors showed similar levels of lipid peroxidation in kidney tissue in AKI and HBO treated groups. Interestingly, the authors did not find any differences in the activity of antioxidative enzymes (SOD, catalase and glutathione peroxidase) among all groups, including a control group. HBO treatment did not improve morphological alterations observed in this study or affect HO-1 activity [53]. Considering that the authors used an identical experimental protocol but a different HBO exposure, there may be a dose-dependent effect in this experimental setting as well.Vrh obrasca.

### 4.4. Effects of Hyperbaric Oxygen Therapy on Cyclosporine-Induced Nephrotoxicity

Cyclosporine A is a calcineurin inhibitor used as an immunosuppressant in transplant patients and for managing autoimmune diseases. However, its clinical use is often restricted due to its nephrotoxic effects, which manifest in two main forms: acute and chronic nephrotoxicity [70]. Acute nephrotoxicity involves renal vasoconstriction triggered by an imbalance in vasoactive substances, resulting in reversible renal dysfunction. Conversely, chronic nephrotoxicity involves persistent vasoconstriction along with structural damage, such as arteriolopathy and tubulointerstitial fibrosis, which are typically irreversible [71].

Renal ischemia in cyclosporine-A-induced nephrotoxicity is mediated by the renin–angiotensin–aldosterone system, wherein angiotensin II, through the activation of AT1 receptors, participates in renal vasoconstriction and promotes renal fibrosis. Established hypoxia resulting from renal vasoconstriction further promotes increased ROS formation, causing cellular injury and promoting cellular death [71]. Although the precise mechanisms of cyclosporine A nephrotoxicity remain incompletely understood, oxidative stress emerges as a pivotal player by inducing endoplasmic reticulum stress and increasing mitochondrial ROS production. Recent studies suggest that cyclosporine-A-induced autophagy might alleviate the deleterious effects of cyclosporine-A-induced endoplasmic reticulum stress, potentially mitigating renal injury [72].

In two presented studies [54,55], the HBO approach involved exposure to 2.5 ATA for 60 min with a single session per day over 4 [54] or 5 days [55]. However, different experimental protocols were used in these studies. Atasoyu et al. applied 25 mg/kg of cyclosporine A for 4 days, concurrently with HBO treatment [54], while Ay et al. administered 15 mg/kg of cyclosporine A for 14 days, with HBO treatment initiated during the last 5 days of the study [55].

In one of the selected studies, where cyclosporine A was administered at a dose of 25 mg/kg for 4 days concurrently with HBO treatment, no protective effects of this treatment were observed. Morphological features of cyclosporine-A-induced nephrotoxicity were similar in groups with or without HBO treatment, with a significant increase in the number of apoptotic cells in proximal tubules [54]. In another study where cyclosporine A was administered at a dose of 15 mg/kg for 14 days with HBO treatment initiated during the last 5 days, HBO attenuated cyclosporine-A-induced oxidative stress in kidney tissue. The authors noted decreased lipid peroxidation and increased SOD and glutathione peroxidase activity after HBO treatment. However, HBO treatment significantly reduced oxidative stress but did not improve serum creatinine levels, though it did reduce serum urea levels [55]. Considering the different experimental designs used in the aforementioned studies, it is possible that the observed differences can be attributed to this. Additionally, it should be noted that although the authors used the same strain of rats, Wistar albino rats, they employed animals of the opposite sex, which may indicate potential gender differences in the effect of HBO treatment.

### 4.5. Effects of Hyperbaric Oxygen Therapy on Gentamicin and Vancomycin-Induced Nephrotoxicity

Vancomycin is a glycopeptide antibiotic used for treating methicillin-resistant Staphylococcus aureus and methicillin-resistant Staphylococcus epidermis infections with important bactericidal role against Streptococcus, Enterococcus, Actinomyces, Clostridium and Eubacterium species. Considering its excellent bactericidal effectiveness and low cost, it is one of the most routinely prescribed antibiotics, accounting for up to 35% of hospitalized patients with infection. The exact pathophysiological mechanisms of vancomycin-induced nephrotoxicity remain unclear, but it is believed that this phenomenon is mediated by intracellular accumulation of the drug in proximal tubular cells followed by induced oxidative stress, complement activation and inflammatory response, mitochondrial dysfunction and cellular death [73].

On the other hand, gentamicin is an aminoglycoside antibiotic, effective against Gram-negative bacterial infections, particularly those caused by Pseudomonas, Proteus, and Serratia species. Gentamicin-induced nephrotoxicity is caused by the accumulation of this drug in renal proximal convoluted tubules that lead to loss of its brush border integrity, ROS formation, reduction in antioxidant defense, subsequent inflammation, acute tubular injury via apoptosis or necrosis and glomerular congestion, resulting in decreased GFR and renal dysfunction [74]. Tubular cytotoxicity is related to apoptosis as well as necrosis of tubular epithelial cells. In the kidney, gentamicin accumulates in proximal tubular epithelial cells. After endocytosis, gentamicin is then transported to lysosomes, the Golgi apparatus, and the endoplasmic reticulum, where it binds to membrane phospholipids, altering their function. Once the concentration of gentamicin in endosomes surpasses a certain threshold, it triggers membrane destruction, leading to the release of its contents, including gentamicin, into the cytoplasm. Moreover, gentamicin disrupts normal mitochondrial function and activates the intrinsic apoptosis pathway. It interferes with the respiratory chain, reducing ATP synthesis and consequently inducing oxidative stress. Also, it is capable of inducing endoplasmic reticulum stress and activating apoptosis by several different mechanisms. It can also inhibit numerous membrane transport proteins, such as the sodium–potassium pump, leading to cell swelling and subsequent necrosis or apoptosis. Morphological damage of the tubules impairs the reabsorption of water and electrolytes, triggering the activation of the tubule-glomerular feedback loop. Following this physiological adaptation, the flow is reduced due to the production of vasoconstrictors as well as the direct effect of gentamicin on vascular cells. Considering that the glomerulus is the first spot that encounters gentamicin, multiple mechanisms are responsible for glomerular toxicity: mesangial contraction and reduced GFR, proliferation of mesangial cells with an increase in apoptosis of those cells, mild morphological enlargement of glomeruli and change in circular shape and density with neutrophil infiltration [75].

Considering three selected studies [56,57,58], in two studies, HBO exposure included exposure to 2.0 ATA for 60 min with one single session per day for 5 [57] or 7 days [56], immediately after the gentamicin [57] or vancomycin [56] injection in Sprague–Dawley rats. Gentamicin nephrotoxicity was induced by intraperitoneal application of 150 mg/kg of gentamicin for 5 consecutive days [57], while vancomycin nephrotoxicity was induced by applying vancomycin at a dose of 500 mg/kg for 7 days [56]. In the third study, gentamicin was applied in a dose of 100 mg/kg, intraperitoneally for 7 days, in Wistar rats, while HBO approach included exposure to 2.5 ATA during 90 min with one single session per day during the same 7 days [58].

Two of the three selected studies did not show beneficial effects of HBO in gentamicin- and vancomycin-induced nephrotoxicity considering present morphological alterations and urea and creatinine serum levels [56,57]. On the other hand, another study that examined the effects of HBO treatment on gentamicin-induced nephrotoxicity showed obvious protective effects through decreased morphological alterations and decreased KIM-1, reflected in improved kidney function and increased levels of creatinine and urea. HBO showed remarkable antioxidant properties, with decreased lipid peroxidation, total oxidant status and improved SOD, glutathione peroxidase activity and total antioxidant status in kidney tissue. The anti-inflammatory effect was reflected through reduced expression of TNF-α and IL-1β in kidney tissue [58]. A possible explanation for the differences in the effects of HBO on gentamicin-induced nephrotoxicity may be the consequence of a different experimental protocol, and a dose-dependent effect of gentamicin, where HBO had no effect when a higher dose of gentamicin was applied. Also, in those studies, different HBO exposure was used, where a beneficial effect was observed in an approach that included longer total and daily exposure, as well as exposure at higher pressure.

### 4.6. Effects of Hyperbaric Oxygen Therapy on Sepsis-Induced Acute Kidney Injury

Sepsis-induced AKI is a common complication of the critically ill patient, associated with unacceptable morbidity and mortality, and carries the risk of developing CKD after recovery. Prevention is difficult because most of the patients, by the time they seek medical support, have already developed AKI. Thus, early recognition and supportive treatment are pivotal to avoid further complications [76]. Although ischemia-reperfusion injury is one of the significant mechanisms, sepsis-induced AKI can occur even in the absence of hemodynamic instability. After the invasion of infectious pathogens, released pathogen-associated molecular patterns (PAMPs) and damage-associated molecular patterns (DAMPs) can bind to Toll-like receptors (TLRs), expressed on the immune cells, endothelial cells, and tubular epithelial cells, and stimulate increased synthesis of proinflammatory cytokines and ROS and promote endothelial activation. Endothelial activation is associated with chemotaxis and amplification of an inflammatory response, with increased vascular permeability and interstitial edema, which further complicates oxygen delivery to tubular epithelial cells and causes hypoxic cell injury [76,77].

Only one study analyzed the effects of HBO in experimental sepsis-induced AKI in Wistar rats. In this study, sepsis was induced by intraperitoneal Escherichia coli injection, while HBO was administered after sepsis induction by using the following: five sessions of HBO treatment were performed in total, with a 6 h interval between sessions. Each session lasted 90 min, with a chamber pressure of 2 ATA. Considering the results of this study, HBO significantly improved kidney function, evaluated by decreased creatinine levels in plasma, and improved GFR and urine output. Also, HBO reduced lipid peroxidation and increased SOD and catalase activity in kidney tissue. Morphological changes in the renal cortex were considerably lower after HBO treatment, but although the degree of congestion slightly decreased, mononuclear cell infiltration was still severe [59]. Like the previous ones, this study confirmed a significant antioxidant effect of HBO. Considering sepsis-induced AKI pathophysiology, and since cellular oxidative injury plays a crucial role in sepsis-induced AKI, it is reasonable to assume that HBO can have a potential beneficial effect in this experimental setting, but further studies are necessary to confirm this.

Although AKI is one of the most common complications, sepsis and septic shock can also cause other serious complications, such as acute respiratory distress syndrome, disseminated intravascular coagulation, mesenteric ischemia, acute liver failure, myocardial dysfunction and multiple organ failure [78]. HBO treatment has been shown to improve survival and reduce complications in experimental animal sepsis models, as well as in clinical studies on patients with necrotizing soft tissue infections [79]. HBO offers several mechanisms that may be beneficial in sepsis, including restoration of mitochondrial function, improved microvascular function and organ perfusion, decreased capillary permeability, improved cytokine profile, direct antimicrobial effects and enhanced antibiotic function [80]. Also, in sepsis, HBO treatment is capable of increasing HO-1 activity, inhibiting NO production and preventing lung damage caused by lipopolysaccharides, reducing inflammatory mediators, modulating NF-κB activity and inhibiting excessive myeloperoxidase production, suppressing bacterial growth in the small intestine, preserving erythrocyte deformability and accelerating free radical acceptor synthesis to mitigate the effects of ROS [81]. On the other hand, clinical data are ambiguous and suggest that HBO therapy, in patients with sepsis, may aggravate existing oxidative stress and contribute to the development of disseminated intravascular coagulation, which is why further clinical studies should be performed in order to elucidate the role of HBO in sepsis [80,81].

### 4.7. Effects of Hyperbaric Oxygen Therapy on Ischemia-Reperfusion Acute Kidney Injury after Kidney Transplantation

AKI is a common feature in kidney transplant recipients, associated with allograft loss and significant mortality [82]. In kidney transplantation, ischemia-reperfusion AKI is an important deleterious factor responsible for graft rejection, chronic allograft nephropathy and loss of kidney transplant function, greatly impacting the long-term survival of the transplanted kidney [60].

One study analyzed the effects of HBO treatment in experimental ischemia-reperfusion AKI after kidney transplantation in Sprague–Dawley rats. In this study, HBO was administered 0, 2, and 4 h after reperfusion, with each session lasting 60 min, with a chamber pressure of 2 ATA. In this study, HBO significantly improved kidney function and decreased creatinine levels 5 h after reperfusion. Also, HBO reduced inflammatory response by decreasing the expression of ICAM-1, VCAM-1 and C3 in kidney tissue. The obtained results were accompanied by a significant improvement in the morphology of the kidney tissue [60]. In isolated kidneys, HBO administration during the cold ischemic phase in organs removed from donors before kidney transplantation is capable of reducing apoptosis, inflammatory cytokine release, and morphological alterations in kidney tissue, as well as KIM-1 expression [83].

## 5. Conclusions

Taking into account the results of conducted experimental studies, as well as the low cost and insignificant adverse effects of HBO, it can be concluded that HBO treatment potentially represents a possible preventive and therapeutic method in ischemia-reperfusion AKI. On the other hand, the results of studies that examined the protective effect of HBO treatment in AKI caused by other etiological factors are inconsistent and still insufficient to draw valid conclusions. Considering the fact that a very small number of studies have been performed so far, it is necessary to plan further experimental studies, in which the new perspective of potential beneficial HBO effects on the pathogenesis, course and outcomes of AKI will be confirmed. Since the data of conducted experimental studies in this experimental setting suggest a dose-dependent effect of HBO treatment, further experimental studies are also necessary to explore the most optimal exposure protocol as well as more precise and detailed molecular mechanisms through which HBO achieves its effects. The fact that some beneficial effects of HBO in the experimental model of post-ischemic AKI are mediated by increased ROS production raises the question related to potential side effects of HBO on organs with normal oxygen supply. This is especially important in kidney-specific HBO delivery regarding the potential protective effects of HBO exposure when it is used as a pretreatment in conditions that carry a high risk for the development of post-ischemic AKI. Animal experiments suggest that HBO delivery in normal renal oxygen supply does not harm renal function or renal morphology [15]. But taking into account the relatively small number of conducted studies performed on animal experimental models, as well as the lack of literature data of HBO effects on organs with normal oxygen supply in the human population, further studies are needed to elucidate the importance of kidney-specific HBO delivery. The results of conducted experimental studies presented in this systematic review may serve as a solid base for future clinical research and potential larger multicentric and multidisciplinary projects focused on clinical confirmation of the obtained results.

## Figures and Tables

**Table 1:** Effects of hyperbaric oxygen preconditioning on acute kidney injury.

Author	Experimental Protocol	HBO Exposure	Main Findings
Gurer et al. [36]	Ischemia-reperfusion injury induced in Sprague–Dawley rats	2.8 ATA, 75 min, 1 single session	HBO significantly decreased lipid peroxidation in kidney tissue and improved renal morphology.
He et al. [37]	Ischemia-reperfusion injury induced in Sprague–Dawley rats	2.5 ATA, 60 min, 2 sessions per day, with total number of 4 sessions	HBO significantly decreased serum urea and creatinine, improved renal morphology, induced expression of HO-1 in kidney tissue.
Kovacevic et al. [38]	Ischemia-reperfusion injury induced in Spontaneously hypertensive rats	2.0 ATA, 60 min, 2 sessions per day, with total number of 4 sessions	HBO improved renal hemodynamics, decreased urea, creatinine, phosphate and KIM-1 levels in plasma, improved renal morphology.
Nesovic Ostojic et al. [39]	Ischemia-reperfusion injury induced in Spontaneously hypertensive and Wistar rats	2.0 ATA, 60 min, 2 sessions per day, with total number of 4 sessions	HBO in both strains improved clearances of urea, creatinine and phosphate, improved renal morphology and increased Bax expression in kidney tissue. In SHR strain, also decreased plasma KIM-1 levels and increased expression of Bcl-2 and HO-1 in kidney tissue.
Kovacevic et. al. [40]	Ischemia-reperfusion injury induced in Spontaneously hypertensive rats	2.0 ATA, 60 min, 2 sessions per day, with total number of 4 sessions	HBO improved clearances of urea, creatinine and phosphate, decreased expression of 4-HNE and NGAL and provoked different HO-1 immunohistochemical expression pattern in kidney tissue.

MDA—malondialdehyde; 4-HNE—4-hydroxy-2-nonenal; HO-1—heme oxygenase—1; SHR—Spontaneously hypertensive rats; KIM-1—kidney injury molecule—1; NGAL—neutrophil gelatinase-associated lipocalin; Bax—Bcl-2-associated protein X.

**Table 2:** Effects of hyperbaric oxygen therapy on acute kidney injury.

Author	Experimental Protocol	HBO Exposure	Main Findings
Solmazgul et al. [44]	Ischemia-reperfusion injury induced in Sprague–Dawley rats	2.5 ATA, 60 min, 1 single session	HBO decreased plasma urea and creatinine, improved renal morphology and decreased neutrophil infiltration in kidney tissue.
Rubinstein et al. [45]	Ischemia-reperfusion injury induced in Sprague–Dawley rats	2.5 ATA, 90 min, 2 sessions per day, with total number of 2 sessions	HBO improved renal hemodynamics and GFR, decreased lipid peroxidation and increased SOD activity in kidney tissue.
Ramalho et al. [46]	Ischemia-reperfusion injury induced in Wistar albino rats	2.5 ATA, 60 min, 2 sessions per day, with total number of 2 sessions	HBO significantly decreased serum creatinine and urea levels, reduced proteinuria and decreased fractional excretions of sodium and potassium, with also improved renal morphology and decreased PCNA expression in kidney tissue, without any effects on macrophage infiltration or HO-1 expression.
Ilhan et al. [47]	Ischemia-reperfusion injury induced in Sprague–Dawley rats	2.5 ATA, 60 min, 1 session per day, with total number of 5 sessions	HBO decreased lipid peroxidation and improved SOD and glutathione peroxidase activity in kidney tissue, improved renal morphology and decreased neutrophil infiltration in kidney tissue.
Migita et al. [48]	Ischemia-reperfusion injury induced in Sprague–Dawley rats	2.5 ATA, 60 min, 1 single session	HBO improved renal morphology, leading to the lower number of TUNEL+ cells and a higher number of Ki67+ cells in the cortex and outer stripe of the medulla.
Ko et al. [49]	Ischemia-reperfusion injury induced in Sprague–Dawley rats	2.4 ATA, 90 min, 1 session per day, with total number of 2 sessions	HBO significantly decreased serum urea and creatinine, improved kidney morphology, decreased oxidative stress (H_2_DCFDA, Nox-1 and Nox-2 levels), increased antioxidative defense (increased NQO 1 and HO–1), decreased inflammation (decreased MMP-9, TNFα, NF-κB, ICAM-1 and F4/80 and CD14-positive cells in kidney tissue), decreased apoptosis (decreased Bax, caspase 3 and cleaved PARP), increased endothelial cell integrity with increased eNOS and CD31 expression and increased CD31 and fibronection-positive cells, increased podocyte integrity with increased ZO-1, E-cadherin, dystrophin and nephrin expression, increased angiogenesis (SDF-1α and VEGF expression), decreased WT-1 and KIM-1 expression in kidney tissue.
Atasoyu et al. [50]	Cisplatin-induced AKI in Wistar albino rats	2.5 ATA, 60 min, 1 session per day, with total number of 7 sessions	HBO significantly decreased serum urea and creatinine, improved their clearances and improved renal morphology.
Aydinoz et al. [51]	Cisplatin-induced AKI in Wistar albino rats	2.5 ATA, 60 min, 1 session per day, with total number of 6 sessions; 2 session per day, with total number of 12 sessions	HBO decreased lipid peroxidation and improved SOD and glutathione peroxidase activity in kidney tissue, especially after two daily sessions. On the other hand, one daily HBO treatment slightly reduced serum urea and creatinine levels and attenuated histopathological injury, while two daily sessions increased creatinine levels and morphological alterations.
Ayvaz et al. [52]	Glycerol-induced AKI in Sprague–Dawley rats	2.5 ATA, 90 min, 1 session per day, with total number of 2 sessions	HBO decreased plasma urea and creatinine levels and increased creatinine clarence, improved renal morphology, decreased lipid peroxidation and increased SOD and catalase activity in kidney tissue, decreased tubular cell apoptosis and decreased PCNA expression.
Cebi et al. [53]	Glycerol-induced AKI in Wistar albino rats	2.4 ATA, 70 min, 2 sessions per day, with total number of 2 sessions	HBO did not have beneficial effects in this experimental setting.
Atasoyu et al. [54]	Cyclosporine-induced nephrotoxicity in Wistar rats	2.5 ATA, 60 min, 1 single session, with total number of 4 sessions	HBO did not have beneficial effects in this experimental setting.
Ay et al. [55]	Cyclosporine-induced nephrotoxicity in Wistar rats	2.5 ATA, 60 min, 1 single session, with total number of 5 sessions	HBO decreased serum urea levels, decreased lipid peroxidation and increased SOD and glutathione peroxidase activity in kidney tissue.
Sabler et al. [56]	Vancomycin-induced nephrotoxicity in Sprague–Dawley rats	2.0 ATA, 60 min, 1 session per day with total number of 7 sessions	HBO did not have beneficial effects in this experimental setting.
Berkovitch et al. [57]	Gentamicin-induced nephrotoxicity in Sprague–Dawley rats	2.0 ATA, 60 min, 1 session per day with total number of 5 sessions	HBO did not have beneficial effects in this experimental setting.
Oztopuz et al. [58]	Gentamicin-induced nephrotoxicity in Wistar rats	2.5 ATA, 90 min, 1 session per day with total number of 7 sessions	HBO decreased serum urea and creatinine, decreased lipid peroxidation and total oxidant status, improved SOD, glutathione peroxidase activity and total antioxidant status in kidney tissue, improved renal morphology and decreased expression of TNF-α, IL-1β and KIM-1.
Edremitlioglu et al. [59]	Sepsis-induced AKI in Wistar albino rats	2.0 ATA, 90 min, 1 session per 6 h, with total number of 5 sessions	HBO significantly decreased plasma creatinine levels, reduced lipid peroxidation and improved cortical SOD and catalase, and medullar catalase activity in kidney tissue, improved GFR, urine output and renal morphology.
Zhao et al. [60]	Ischemia-reperfusion AKI after kidney transplantation in Sprague–Dawley rats	2.0 ATA, 60 min, 1 session per 2 h, with total number of 3 sessions	HBO significantly decreased serum creatinine levels, decreased immunohistochemical expression of ICAM-1, VCAM-1, and C3 as well as ICAM-1 and C3 mRNA in kidney tissue and improved renal morphology.

SOD—superoxide dismutase; GFR—glomerular filtration rate; PCNA—proliferation cell nuclear antigen; IL-1β—interleukin 1 beta; ICAM-1—intercellular adhesion molecule 1; VCAM-1—vascular cell adhesion molecule 1; TUNEL—terminal deoxynucleotidyl transferase dUTP nick end labeling; TNF α—Tumor necrosis factor alpha; H2DCFDA—2′,7′-dichlorodihydrofluorescein diacetate; Nox 1/2—NADPH oxidase 1/2; NQO 1—NAD(P)H quinone oxidoreductase; HO-1—heme oxygenase—1 MMP 9—matrix metalloproteinase 9; NF—kβ—nuclear factor kappa-light-chain-enhancer of activated B cells; CD14—cluster of differentiation 14; PARP—poly (ADP-ribose) polymerase; eNOS—endothelial nitric oxide synthase; ZO1—Zonula Occludens 1 protein; SDF-1α—stromal-cell-derived factor-1α; VEGF—vascular endothelial growth factor; WT1—Wilms tumor protein type 1; KIM-1—kidney injury molecule-1.

## Data Availability

The data presented in this study are available within the article.
